# Comparative Transcriptome Analysis Reveals Gene Expression Differences in Eggplant (*Solanum melongena* L.) Fruits with Different Brightness

**DOI:** 10.3390/foods11162506

**Published:** 2022-08-19

**Authors:** Aidong Zhang, Qianru Huang, Jianyong Li, Weimin Zhu, Xiaohui Liu, Xuexia Wu, Dingshi Zha

**Affiliations:** 1 Shanghai Key Laboratory of Protected Horticultural Technology, Horticultural Research Institute, Shanghai Academy of Agricultural Sciences, Shanghai 201403, China; 2 College of Life Science, Shanghai Normal University, Shanghai 201418, China; 3 Shanghai Agricultural Technology Extension Service Center, Shanghai 201103, China; 4 College of Fisheries and Life Science, Shanghai Ocean University, Shanghai 201306, China

**Keywords:** eggplant, brightness, wax, transcriptome analysis, KCS

## Abstract

Fruit brightness is an important quality trait that affects the market value of eggplant. However, few studies have been conducted on eggplant brightness. In this study, we aimed to identify genes related to this trait in three varieties of eggplant with different fruit brightness between 14 and 22 days after pollination. Using RNA-Seq Gene Ontology and Kyoto Encyclopedia of Genes and Genomes enrichment analyses, we found that wax- and cutin-related pathways and differentially expressed genes displayed significant differences among different development stages and varieties. Scanning electron microscopy revealed that the wax layer was thinner in ‘30-1’ and ‘QPCQ’ than in ‘22-1’. Gas chromatography-mass spectrometry analysis revealed that wax content was significantly lower in ‘30-1’ than in ‘22-1’, which indicated that wax may be an important factor determining fruit brightness. We further identified and analyzed the KCS gene family, which encodes the rate-limiting enzyme of FA elongation in wax synthesis. The results provide an insight into the molecular mechanisms of fruit brightness in eggplants and further eggplant breeding programs.

## 1. Introduction

The eggplant (*Solanum melongena* L.) is a solanaceous crop that is grown worldwide. Additionally, its fruits are an important component of the human diet, providing vitamins and active phytochemical compounds. Fruit brightness is a highly valuable external quality trait that affects the market value of eggplants. Compared with dull fruit, the bright appearance of glossy fruit attracts consumers [1,2,3].

Previous studies on fruit brightness have mainly focused on other crops such as cucumber, tomato, *Arabidopsis*, and navel orange. Early genetic studies have shown that the dull fruit skin phenotype (D) is dominant over the glossy fruit skin phenotype (d) in cucumber [4,5]. Yuan et al. [6] found that *D* was mapped between two markers, ME23EM4 and CS15, by constructing a genetic linkage map using 224 recombinant inbred lines (RILs). Miao et al. [7] constructed a new genetic linkage map using 148 RILs, where the *d* gene was found to be mapped between two simple sequence repeat (SSR) markers, SSR06003 and SSR15818, on chromosome 5. Yang et al. [5] preliminarily mapped markers SCZ69 and SSR16203 by combining bulked segregant analysis and 11 polymorphic molecular markers on chromosome 5. For further high-resolution mapping of *D/d* genes by increasing the F2 population, they identified *Csa016880* or *Csa016887* as candidate gene *D* in cucumbers. In tomatoes, cutin deficiency has been reported to contribute to fruit glossiness [8,9,10]. However, Petit et al. [11] found that cuticle architecture was responsible for fruit glossiness by analyzing ethyl methanesulfonate mutants in tomatoes. In *Arabidopsis*, stem glossiness is generally caused by a reduction in wax load or an alteration of specific wax compounds [12]. A bud mutation in *Citrus sinensis* (L.) Osbeck cv. ‘Newhall’ branches generates glossier fruits than those of unmutated branches. The wax load of glossy fruits is significantly lower than that of original fruits throughout the development stage [13]. These studies have shown that fruit brightness is closely associated with the cuticle.

The cuticle is an extracellular layer that covers the surface of aerial plant organs. The cuticle is composed of cutin that is embedded with polysaccharides, filled with intracuticular wax, and covered with epicuticular waxes [14]. Cutin is a polyester of hydroxy and epoxy fatty acids [15], whereas wax is composed of very-long-chain fatty acids (VLCFAs) and their derivatives, such as ketones, alkanes, aldehydes, alcohols, and esters [11]. The synthesis of cutin monomers begins with the synthesis of long-chain fatty acids (FAs) in plastids. The long-chain FAs are then transferred to the cytoplasm, where they undergo a series of modifications [16]. The wax synthesis also begins with the synthesis of long-chain FAs. FAs are further elongated to form VLCFAs by FA elongases (FAE), which include β-ketoacyl-CoA synthetase (KCS), β-hydroxyacyl-coenzyme A dehydratase (HCD), β-ketoacyl-coenzyme A reductase (KCR), and trans-2,3-enoyl-coenzyme A reductase (ECR). The biosynthesis of VLCFA aliphatic compound derivatives mainly includes the following two pathways: acyl reduction and decarbonylation. In the acyl reduction pathway, VLCFAs generate the corresponding primary alcohol and wax ester. In the decarbonylation pathway, VLCFAs generate aldehydes, alkanes, secondary alcohols, and ketones.

In our research, we aimed to identify brightness-related genes using three eggplant varieties with the following different levels of brightness: ‘22-1’, which displays a dull peel, and ‘30-1’ and ‘QPCQ-1’, which display glossy peels. Differentially expressed genes (DEGs) involved in wax biosynthesis at different stages of fruit development were identified. These results further explain the mechanism of cuticle formation and provide valuable data for the further study of the underlying mechanisms regulating brightness in eggplants.

## 2. Materials and Methods

### 2.1. Plant Materials

The ‘22-1’, ‘30-1’, and ‘QPCQ’ are all breeding lines selected by the Shanghai Academy of Agricultural Sciences (SAAS), Shanghai, China. In brief, ‘Guangdong green eggplant’ and ‘765 long eggplant’ were crossed. Their offspring were self-crossed for several generations to obtain ‘22-1’ and ‘30-1’ varieties. ‘QPCQ’ was local material collected from farmers. The three varieties were grown at the Horticultural Research Institute of SAAS. To ensure consistent fruit development, all fruits were artificially pollinated on the day of flowering and harvested after between 14 and 22 days. The peel of the fruits was cut off and frozen in liquid nitrogen. Three replicates of each variety were collected and stored at −80 °C until use.

Fruit peel brightness was measured using an intelligent gloss meter (3nh, Shenzhen, China) according to the operating manual. Briefly, the “power” button was pushed to turn on the instrument and “calibration” was selected from the main menu to enter the calibration interface. The instrument was placed in the calibration box for calibration. After completion of the calibration, the measurement “basic mode” was selected, the measurement hole was aligned with the fruits to be measured, and “measurement” was clicked to obtain the value. Fruits were measured in the field, and each variety was measured with three replications.

### 2.2. RNA Extraction, Library Construction, and Illumina Sequencing

Total RNA was extracted using the MiniBEST Universal RNA Extraction Kit (TaKaRa, Tokyo, Japan) according to the manufacturer’s instructions. Total RNA was quantified using a NanoDrop spectrophotometer (Thermo Fisher Scientific, Waltham, MA, USA). The A260/280 ratios of all samples were above two. The 28S/18S ratio and RNA integrity number (RIN) values were determined using an Agilent 2100 system (Agilent, Santa Clara, CA, USA) [17]. The mRNA was purified from total RNA using poly T oligo-attached magnetic beads; an interrupting reagent was added to interrupt the mRNA and produce short fragments. The interrupted mRNA fragments were used as a template to synthesize one-strand cDNA with a six-base random primer. Subsequently, a two-strand synthesis reaction system was prepared to synthesize double-stranded cDNA. The purified double-stranded cDNA was repaired, poly A-tailed, and ligated with sequencing adapters. PCR amplification was performed after fragment size selection. The constructed libraries were qualified using an Agilent 2100 Bioanalyzer (Agilent) and sequenced using an Illumina HiSeq 2500. Sequencing generated 100-bp paired-end reads as raw reads. All of the generated raw sequencing reads were filtered to remove low-quality poly-N and low-quality reads using the software SOAPnuke (BGI). After filtering, the remaining reads were termed ‘clean reads’. The data have submitted to NCBI SRA database with accession number PRJNA721241.

### 2.3. RNA-Seq Data Analyses and DEG Identification

The clean reads were mapped to the eggplant reference genome (http://eggplant.kazusa.or.jp/ accessed on 27 June 2022) by hisat2 (hierarchical indexing for spliced alignment of transcripts) [18], with default parameters. The results of the comparison between the clean reads and the reference genome were stored in binary ‘bam’ files. The expression value from the fragments per kilobase per million mapped (FPKM) method was quantified using the cufflinks [19]. When calculating the difference in gene expression, htseq-count [20] was used to obtain the number of reads in each sample. The estimateSizeFactors function of the DESeq (2012) R package [21] was used to standardize the data, and the nbinomTest function was used to calculate the *p*-value and fold-change value of the different samples. *p* ≤ 0.05 and |log2 fold-change| ≥ 1 were set as thresholds for significantly differential expression.

### 2.4. Annotation and Classification of DEGs

Gene ontology (GO) annotation was performed using the Blast2GO program [22] and GO functional classification with a Pearson chi-squared test was performed using Web Gene Ontology Annotation Plot (WEGO) [23]. The DEGs were mapped to GO terms according to the analyses, and the number of DEGs in each term was calculated [17]. DEGs were mapped into the Kyoto Encyclopedia of Genes and Genomes (KEGG) metabolic pathway database and annotated using BLASTX. A hypergeometric test was applied to the results of GO and KEGG enrichment analyses to identify significant GO terms, enriched metabolic pathways, or signal transduction pathways in DEGs compared with the whole genome background. An adjusted *p*-value of ≤0.05 was used to define significantly enriched GO terms and KEGG pathways [24].

### 2.5. Validation of Gene Expression Profile by qRT-PCR

An Evo M-MLV RT kit (Accurate Biotechnology, Changsha, China) was used to synthesize cDNA from total RNA according to the manufacturer’s instructions. qRT-PCR was performed on an ABI 7500 (Applied Biosystems, Waltham, MA, USA) using a 20-μL reaction mixture, containing 10 μL Hieff^®^ qPCR SYBR Green Master Mix (Low Rox Plus), 0.4 μL of each primer (0.2 μM final concentration), and 2 μL cDNA template, which was diluted four times with ddH_2_O. The PCR amplification procedure consisted of denaturation at 95 °C for 5 min, followed by 40 cycles of denaturation at 95 °C for 10 s, annealing, and extension at 60 °C for 30 s. Transcript levels were calculated using the 2−ΔΔCt method [25]. The *SmEF1a* gene (*Sme2.5_01406.1_g00001.1*) was used as an internal control. Three replicates were performed for the entire experiment. The primers used are listed in Table S1.

### 2.6. Cutin Monomer and Wax Analysis

Cuticular waxes of eggplant were extracted from 50 cm^2^ peels. The peels were dipped into 15 mL of chloroform for 5 min. Subsequently, 100 µg docosane was added to the solution as internal standard. Extracts were dried under moderate nitrogen flux and lipids were derivatized 10 min at 100 °C by adding 100 pyridine and 100 µL of BSTFA (derivatization agent). After cooling down to room temperature, extracts were dried with nitrogen to a constant weight. Further, 500 µL of n-hexane was added to resuspend the sample and draw all samples through a 0.22-µm membrane. Wax was then analyzed and quantified using gas chromatography-mass spectrometry (GC-MS). Cutin monomer analyses were conducted as previously described [9].

### 2.7. Cryo-SEM

Fruit peels at different development stages were cut off and freeze-fixed using liquid nitrogen. Samples were then transferred to a vacuum-sputtering instrument for fracture under freezing conditions to expose their fresh fracture surface. Sublimation was carried out according to the condition of the sample to prevent the surface from being wrapped in ice. The sample preparation was completed after conductive spraying. The samples were placed on the cold stage of a scanning electron microscope (SEM, Hitachi S-4800, Tokyo, Japan) for observation and photo capture. The thickness of cuticle was measured from the SEM photos using Image-Pro plus 6.0 software (Media Cybernetics, Silver Springs, MD, USA).

### 2.8. Identification of KCS Family in Eggplant

An HMM [26] analysis and a simple modular architecture research tool (SMART) [27] were used to identify KCS protein families in the different varieties of eggplant. The HMM profile was downloaded from the Pfam database (http://pfam.xfam.org/ (accessed on 27 June 2022)) to obtain the Pfam serial number (PF08392.12). The hmmsearch function in the HMMER software was used to detect domains contained in the target protein sequence with an e-value ≤ 1 × 10^−3^. The results of HMMER sequence alignment were screened to remove protein sequences whose alignment length was less than 45% of the domain length of the HMM model while retaining the longest alternative splicing sequence. All non-redundant protein sequences were further analyzed using SMART (http://smart.embl-heidelberg.de/ (accessed on 27 June 2022)) for examination, and the same genes were confirmed as family members.

### 2.9. Phylogenetic Analysis

The identified 19 SmKCS protein sequences were aligned using ClustalX software with default parameters [28]. The phylogenetic tree was constructed using the neighbor-joining method in MEGA7.0 with 1000 bootstrap tests [29].

### 2.10. Structure and Motif Analysis

Information on gene location and exon–intron structures was acquired from the reference genome annotation files. The MEME program (http://alternate.meme-suite.org/tools/meme (accessed on 27 June 2022)) was used to identify the conserved motifs of the KCS sequence, while the maximum motif search value was set at 15 with an optimum motif width of 10–100 amino acid residues.

### 2.11. Data Analysis

SPSS software (IBM, Armonk, NY, USA)was used for data analysis and Origin 9.0 (OriginLab, Northampton, MA, USA) was used for drawing. All data are presented as the mean ± standard deviation (SD) of three biological replicates. Tukey’s at *p* = 0.01 or *p* = 0.05 was used to identify the significant differences.

## 3. Results

### 3.1. Identification of Three Independent Eggplant Varieties with Different Fruit Brightness

**Figure 1:**
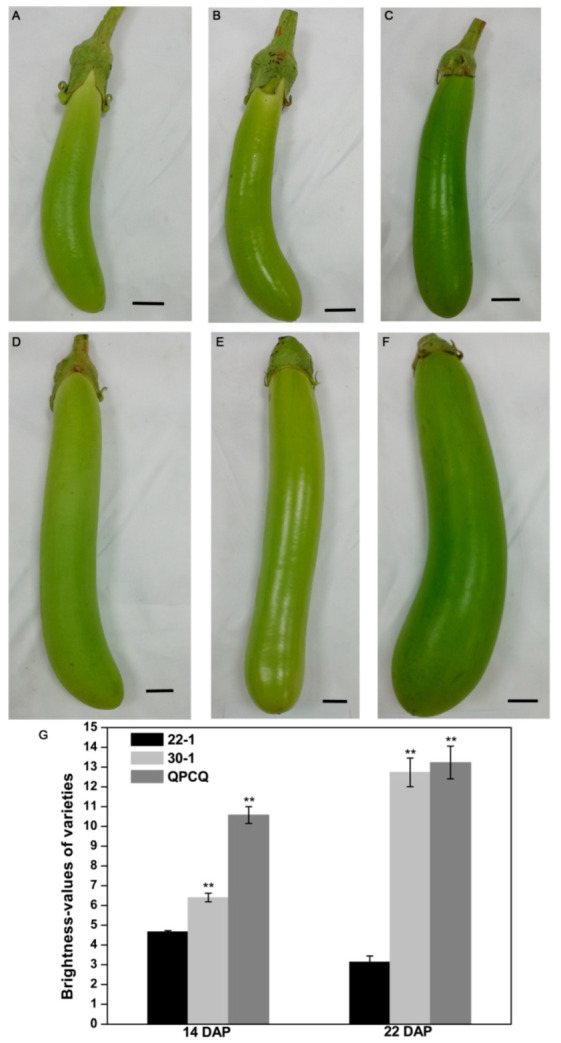
Eggplant fruits with glossy and dull peel at different development stages. The 14-day-pollinated fruits of 22-1 (**A**), 30-1 (**B**), and QPCQ (**C**). The 22-day-pollinated fruits of 22-1 (**D**), 30-1 (**E**), and QPCQ (**F**). Scale bar = 20 mm. (**G**) Brightness values of the three varieties at 14 DAP and 22 DAP. Three fruits of each variety were used to measure brightness values. Asterisks indicate a significant difference of 30-1 and QPCQ against 22-1 in brightness values (** *p* < 0.01).

To explore the differences in fruit brightness in S. melongena, we selected varieties with dull and glossy peels by visual evaluation and gloss meter measurements. The fruit brightness values of varieties ‘30-1’ and ‘QPCQ’, both of which display a glossy peel, were 6.40 and 10.57 on the 14th day after pollination (DAP), respectively, which represented the fruit expansion period, and 12.73 and 13.23 on the 22nd DAP, respectively, which represented the fruit maturation period. The brightness value of variety ‘22-1’ (dull peel) was 4.67 and 3.13 on the 14th and 22nd DAP, respectively (Figure 1).

### 3.2. Fruit Transcriptome Sequencing and Analyses

To further illustrate the molecular mechanisms of fruit brightness in the three eggplant varieties, RNA-Seq libraries were generated with fruit peels of 14 and 22 DAP ‘22-1’, ‘30-1’, and ‘QPCQ’. The libraries were sequenced using an Illumina HiSeq 2500. An overview of the RNA-Seq reads derived from the 18 libraries is presented in Table S2. Approximately 52,512,931 and 50,084,466 raw reads with 7,876,939,600 and 7,512,669,900 bases were generated from the 22-14 (14 DAP fruit peels of ‘22-1’) and 22-22 (22 DAP fruit peels of ‘22-1’) libraries, respectively. Approximately 48,757,086 and 48,757,086 raw reads with 7,313,562,900 and 7,539,632,800 bases were generated from 30-14 (14 DAP fruit peels of ‘30-1’) and 30-22 (22 DAP fruit peels of ‘30-1’), respectively. Approximately between 49,653,755 and 51,810,549 raw reads with 7,448,063,200 and 7,771,582,300 bases were generated from QPCQ-14 (14 DAP fruit peels of ‘QPCQ’) and QPCQ-22 (22 DAP fruit peels of ‘QPCQ’). The Q20 and Q30 of the reads (the proportion of the number of bases with a quality value greater than 20 and 30, respectively, compared to the total number of bases in the raw reads) in these data were over 97% and 92%, respectively (Table S2).

After removing the adapter sequences, poly-N, and low-quality reads from the raw data, the quality of the clean data was assessed. Approximately 48,891,631 (22-14) and 46,712,450 (22-22) clean reads were generated for the ‘22-1’ variety. Approximately 45,530,109 (30-14) and 45,530,109 (30-22) clean reads were generated for the ‘30-1’ variety. Finally, approximately 46,284,605 (QPCQ-14) and 48,232,454 (QPCQ-22) clean reads were generated for the ‘QPCQ’ variety (Table S3). The clean reads were then mapped to the reference genome of the eggplant. In total, 85.03%, 85.24%, 85.15%, 84.48%, 85.02%, and 86.03% of clean reads from 22-14, 22-22, 30-14, 30-22, QPCQ-14, and QPCQ-22, respectively, were mapped to the genome, including 81.98%, 81.12%, 81.94%, 80.32%, 81.90%, and 81.56% uniquely mapped reads, respectively (Table S3).

### 3.3. Prediction of New Transcripts

New transcripts exhibited no annotation information in the reference genome. These new transcripts may have been new splicing subtypes of known genes or new transcripts of unknown genes. In this study, 30,726 new transcripts were detected, including 26,158 coding transcripts and 4568 noncoding transcripts (Table S4).

### 3.4. Single Nucleotide Polymorphisms and INDEL Detection

Single nucleotide polymorphisms (SNPs) are DNA sequence changes caused by single nucleotide changes, resulting in genome diversity among species or individuals. An average of 53,391 SNPs were found in the 22-1, 30-1, and QPCQ samples compared to that in the reference genome (Table 1). The distribution of SNPs was mainly focused on the exons, introns, and intergenic areas (Figure S1). An insertion–deletion (INDEL) is the insertion or deletion of small fragments of less than 50 bp in the sample relative to the reference genome. Similar to that of SNPs, the distribution of INDELs was also concentrated in exons, introns, and intergenic areas (Figure S4).

### 3.5. Analyses of DEGs

**Figure 2:**
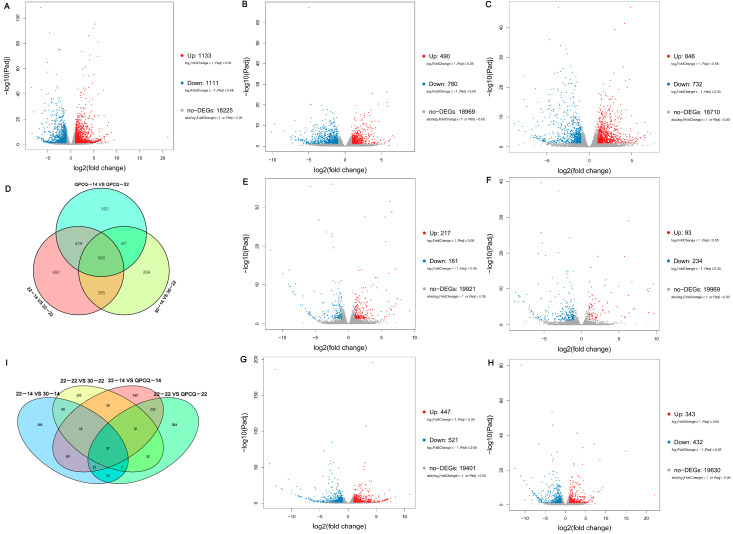
Differentially expressed gene (DEG) analyses across the different eggplant groups. Volcano plots of DEGs in groups 22-22 vs. 22-14 (**A**), 30-22 vs. 30-14 (**B**), QPCQ-22 vs. QPCQ-14 (**C**), 30-14 vs. 22-14 (**E**), 30-22 vs. 22-22 (**F**), QPCQ-14 vs. 22-14 (**G**), and QPCQ-22 vs. 22-22 (**H**). Red dots, blue dots, and gray dots indicate upregulated genes, downregulated genes, and not DEGs, respectively. (**D**) Venn diagram of DEGs in different developmental stages of the three eggplant varieties. (**I**) Venn diagram of DEGs among the different eggplant varieties.

The FPKM fragments method [30] was used to calculate the transcript abundance of genes among the different samples. DEGs were identified by setting a threshold of |log2 fold-change | > 1 and *p* < 0.05. As shown in Figure 2A–C, 1133 upregulated genes and 1111 downregulated genes were identified in group 22-22 compared to that in 22-14. A total of 490 upregulated genes and 780 downregulated genes were identified in group 30-22 compared to that in 30-14. A total of 846 upregulated genes and 732 downregulated genes were identified in group QPCQ-22 compared to that in QPCQ-14. Among these, 580 genes were commonly differentially expressed among the three groups (Figure 2D). There were 398 DEGs (217 upregulated genes and 181 downregulated genes) in 30-14 compared to that in 22-14 and 327 DEGs (93 upregulated genes and 234 downregulated genes) in 30-22 compared to that in 22-22. There were 968 DEGs (447 upregulated genes and 521 downregulated genes) in QPCQ-14 compared to that in 22-14 and 775 DEGs (343 upregulated genes and 432 downregulated genes) in QPCQ-22 compared to that in 22-22, respectively (Figure 2E–H). After comparing the DEGs of the different varieties, we found 37 genes that were differentially expressed in these four groups (Figure 2I).

### 3.6. GO Functional Enrichment Analyses of DEGs

**Figure 3:**
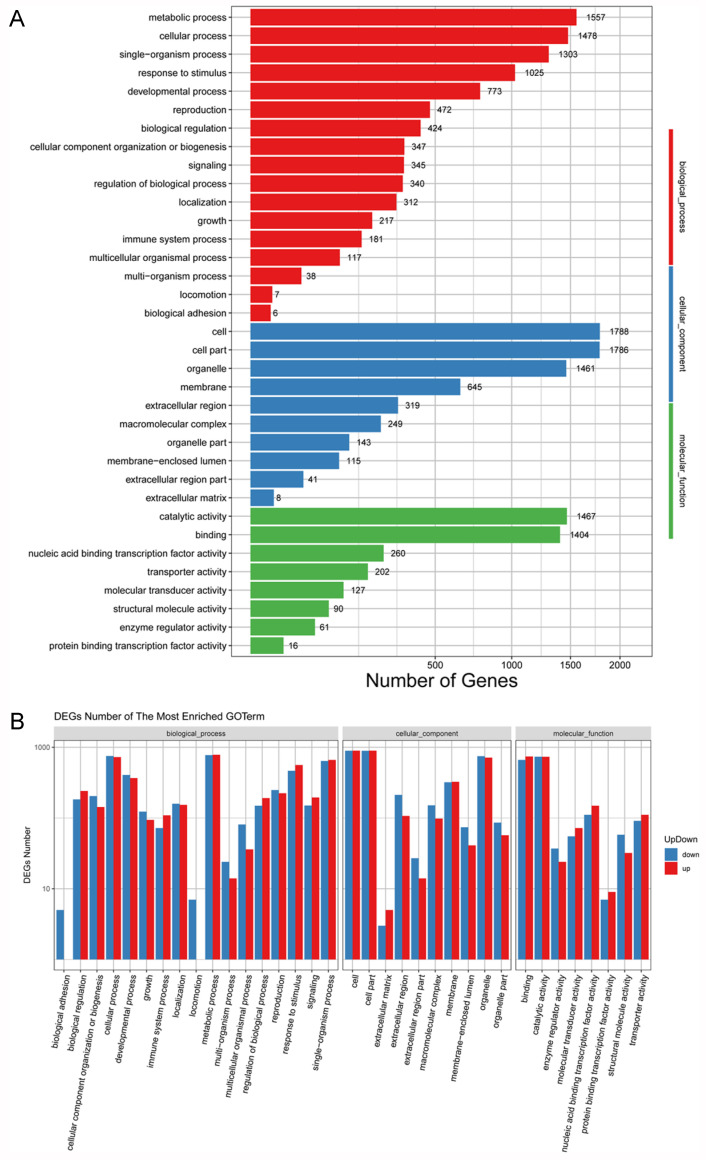
GO functional enrichment analyses of the differentially expressed genes (DEGs) in group 22-22 vs. 22-14. (**A**) GO functional enrichment analyses of the DEGs in group 22-22 vs. 22-14. (**B**) GO functional enrichment analyses of up- and downregulated genes in group 22-22 vs. 22-14.

To further illustrate the potential function of the DEGs, their function nal classes were evaluated using GO enrichment analyses. GO is classified into the following three major functional categories: molecular function, cellular component, and biological process. As shown in Figure 3A and Figure S3A–F, the DEGs were mainly clustered in the metabolic processes, cellular processes, single-organism processes, responses to stimuli, and developmental processes of the biological process category. Cell, cell part, organelle, and membrane dominated the cellular component category in all groups. The most common terms in the molecular function category were catalytic activity, binding, nucleic acid binding transcription factor activity, transporter activity, and molecular transducer activity. To further analyze the classification of upregulated and downregulated DEGs, we found that the most significantly different terms of the three GO categories in the seven groups were consistent with the previous DEG classification (Figure 3B and Figure S3G–L).

### 3.7. KEGG Pathway Database Enrichment Analyses of DEGs

**Figure 4:**
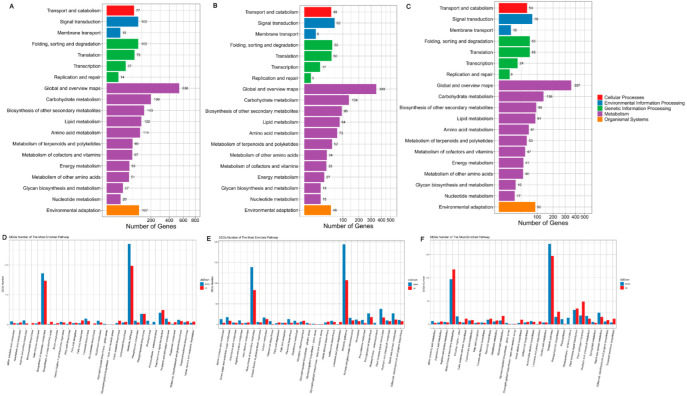
KEGG pathway classification of differentially expressed genes (DEGs) in groups 22 DAP vs. 14 DAP. KEGG plots of groups 22-22 vs. 22-14 (**A**), 30-22 vs. 30-14 (**B**), and QPCQ-22 vs. QPCQ-14 (**C**). Top 30 pathways and up-/downregulated DEGs in these pathways in groups 22-22 vs. 22-14 (**D**), 30-22 vs. 30-14 (**E**), and QPCQ-22 vs. QPCQ-14 (**F**).

**Figure 5:**
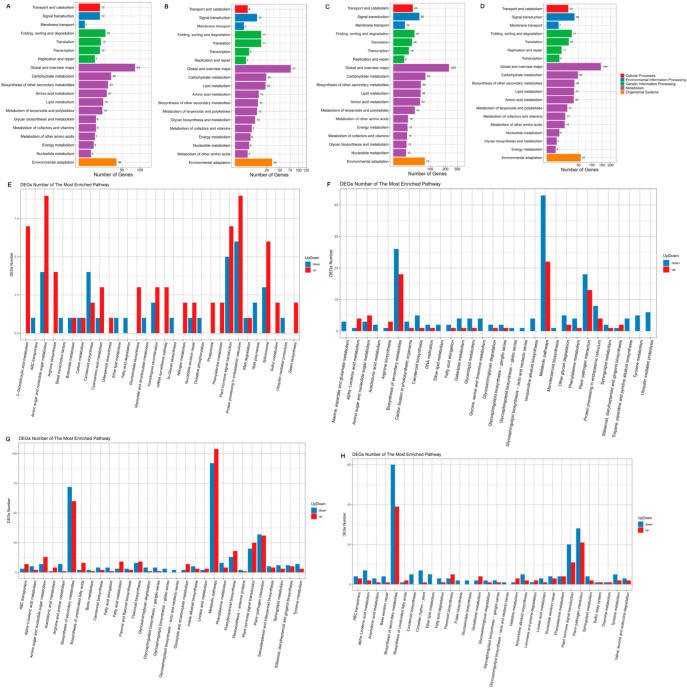
KEGG pathway classification of DEGs among different eggplant varieties. KEGG plots of groups 30-14 vs. 22-14 (**A**), 30-22 vs. 22-22 (**B**), QPCQ-14 vs. 22-14 (**C**), and QPCQ-22 vs. 22-22 (**D**). Top 30 pathways and up-/downregulated DEGs in these pathways in groups 30-14 vs. 22-14 (**E**), 30-22 vs. 22-22 (**F**), QPCQ-14 vs. 22-14 (**G**), and QPCQ-22 vs. 22-22 (**H**).

According to the results of the DEG analysis, we performed KEGG pathway classification and enrichment analyses. KEGG pathways are divided into the following seven categories: cellular processes, environmental information processing, genetic information processing, human diseases (animals only), metabolism, organismal systems, and drug development. The DEGs in the seven groups were enriched in cellular processes, environmental information processing, genetic information processing, metabolism, and organismal systems. Among them, metabolism accounted for the largest portion (Figure 4A–C and Figure 5A–D).

### 3.8. DEGs Related to Cutin and Wax in Eggplants of Different Brightness

After mapping the upregulated and downregulated DEGs in the KEGG database, we analyzed the top 30 pathways that were most significantly enriched. We found that several pathways related to the biosynthesis, metabolism, and transport of cutin, and wax accounted for a significant portion. In a previous study, cutin and wax affected fruit brightness [13]. We further analyzed the expression pattern of genes related to cutin and wax.

**Figure 6:**
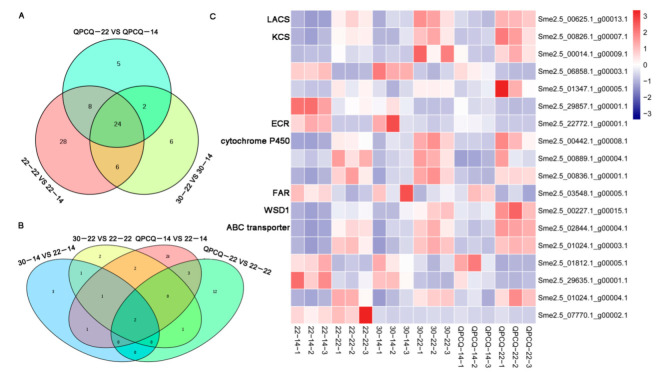
Differentially expressed genes (DEGs) related to cutin and wax in the three varieties of eggplant. Venn plots of 22 DAP vs. 14 DAP in three varieties (**A**), groups 30 vs. 20 and QPCQ vs. 20 (**B**). (**C**) Heat map of DEGs related to cutin and wax in eggplants of different brightness.

In the 22-22 vs. 22-14 group, four pathways, ‘biosynthesis of unsaturated fatty acids’, ‘fatty acid biosynthesis’, ‘fatty acid elongation’, and ‘fatty acid metabolism’, displayed significant enrichment. The ‘fatty acid degradation’ and ‘fatty acid elongation’ pathways were significantly enriched in the 30-22 vs. 30-14 group. ‘Cutin, suberine, and wax biosynthesis’ and ‘fatty acid elongation’ were significantly enriched in the QPCQ-22 vs. QPCQ-14 group (Figure 4D–F). Subsequently, we selected all pathways related to cutin and wax in these three groups. As shown in Table 2, 66, 38, and 39 DEGs involved in ‘biosynthesis of unsaturated fatty acids’, ‘cutin, suberine, and wax biosynthesis’, ‘fatty acid biosynthesis’, ‘fatty acid degradation’, ‘fatty acid elongation’, ‘fatty acid metabolism’, and ‘ABC transporters’ pathways were selected for further analysis in the three groups, respectively. After Venn plot analysis, 24 DEGs were common in the brightness of the three groups and displayed similar expression patterns (Figure 6A). A total of sixteen DEGs were upregulated and eight DEGs were downregulated in the three groups (Table 3). Four genes encoding KCSs, the rate-limiting step enzymes in fatty acid elongation, were differentially expressed across the three groups. *Sme2.5_22772.1_g00001.1*, which encodes ECR, was downregulated in all three groups. Three genes belonging to the cytochrome P450 family were upregulated. *Sme2.5_03548.1_g00005.1*, which encodes fatty acyl-CoA reductase (FAR), was downregulated, as was *Sme2.5_00227.1_g00015.1*. Five DEGs belonging to the ABC transporter family were significantly differentially expressed among the three groups. Three of these genes were upregulated, whereas two were downregulated (Figure 6C and Table 3).

When the KEGG pathway categories of group 30-14 were compared to that of 22-14, the ‘ABC transporter’ and ‘fatty acid degradation’ pathways showed significant differences. The ‘fatty acid elongation’ pathway was significantly enriched in group 30-22 compared to that of group 22-22. Four pathways, ‘ABC transporter’, ‘biosynthesis of unsaturated fatty acids’, ‘fatty acid elongation’, and ‘fatty acid metabolism’, were significantly enriched in the QPCQ-14 vs. 22-14 group, while three pathways, ‘ABC transporter’, ‘biosynthesis of unsaturated fatty acids’, and ‘fatty acid degradation’, were significantly enriched in the QPCQ-22 vs. 22-22 group (Figure 5E–H). After selecting all pathways related to cutin and wax, we found that eight and nine DEGs were enriched in the 30-14 vs. 22-14 and 30-22 vs. 22-22 groups, respectively. In the QPCQ-14 vs. 22-14 and QPCQ-22 vs. 22-22 groups, the expression levels of 33 and 18 DEGs were significantly different, respectively (Figure 6B). To further compare the differences among the three eggplant varieties, we created a Venn plot. Two genes (*Sme2.5_07770.1_g00002.1* and *Sme2.5_29857.1_g00001.1*) were commonly downregulated in the four groups (Figure 6B,C). *Sme2.5_07770.1_g00002.1* encodes ABC transporter C family member 8, while *Sme2.5_29857.1_g00001.1* encodes KCS.

### 3.9. DEGs Identified as Transcription Factors in Eggplants of Different Brightness

**Figure 7:**
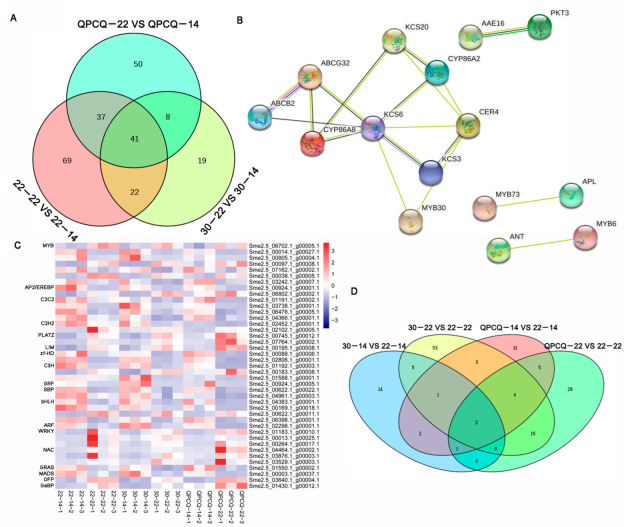
Differentially expressed genes (DEGs) identified as transcription factors (TFs) in eggplants of different brightness. (**A**) Venn plot of groups 22-22 vs. 22-14, 30-22 vs. 30-14, and QPCQ-22 vs. QPCQ-14. (**B**) Interaction net among TFs and cuticle-related genes at different eggplant development stages. (**C**) Heat map of 41 TFs at different eggplant development stages. (**D**) Venn plot of groups 30-14 vs. 22-14, 30-22 vs. 22-22, QPCQ-14 vs. 22-14, and QPCQ-22 vs. 22-22.

After predicting the encoding abilities of DEGs, we found 1172 DEGs, which were classified into 60 transcription factor (TF) families, in all groups (Figure S4). There were 169, 90, and 136 TFs in the 22-22 vs. 22-14, 30-22 vs. 30-14, and QPCQ-22 vs. QPCQ-14 groups, respectively. In total, 41 TFs were commonly identified in the three eggplant varieties (Figure 7A). Among them, seven genes belonged to the MYB family, and two genes belonged to the AP2/EREBP family (Figure 7C). String websites were used to analyze the potential interactions among these nine TFs and the previously screened cuticle-related genes. MYB30 (Sme2.5_00097.1_g00008.1) may participate in cuticle formation by interacting with KCS6 (Sme2.5_29857.1_g00001.1) and CER4 (Sme2.5_03548.1_g00005.1) (Figure 7B). In the 30-14 vs. 22-14, 30-22 vs. 22-22, QPCQ-14 vs. 22-14, and QPCQ-22 vs. 22-22 groups, there were 25, 83, 31, and 56 DEGs, respectively. After Venn plot analysis, we found that only two genes (*Sme2.5_02826.1_g00006.1* and *Sme2.5_04916.1_g00002.1*) were commonly identified in these groups (Figure 7D).

### 3.10. Cuticle Condition of Eggplants of Different Brightness

**Figure 8:**
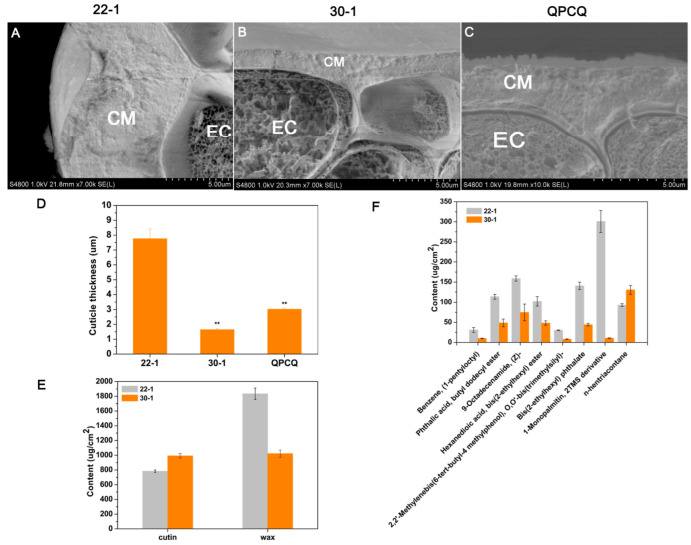
Cuticle condition of eggplant varieties of different brightness. Scanning electron microscopy images of 22 DAP fruit peels from ’22-1’ (**A**), ’30-1’ (**B**), and ‘QPCQ’ (**C**). CM, cuticular membrane; EC, epidermal cell. (**D**) Cuticle thickness statistics of 22 DAP fruit peels from ’22-1’, ’30-1’, and ‘QPCQ’. (**E**) Cutin and wax contents of 22 DAP fruits from ’22-1’ and ’30-1’. (**F**) Wax components with significant differences between ’22-1’ and ’30-1’. Asterisks indicate a significant difference in cuticle thickness (** *p* < 0.01).

Using the analyses mentioned above, we identified several pathways and genes related to cutin and wax that displayed significant differences among the different eggplant varieties. We hypothesized that the cuticle of the eggplant fruits may have contributed to the brightness. To verify our hypothesis, we used SEM to observe the fruit peels of the different brightness varieties of eggplant. As shown in Figure S5, the cuticle of ‘22-1’ displayed relatively more irregular and circular-shaped, dome-like structures than those of 30-1 and QPCQ. After further analysis of the side structure of the peels using SEM, we found that the cuticular membrane of ‘22-1’ was thicker than that of ‘30-1’ and ‘QPCQ’ (Figure 8A–C). The average cuticle thickness of ‘22-1’ reached 7.76 μm, whereas that of ‘30-1’ and ‘QPCQ’ reached only 1.65 μm and 3.02 μm, respectively (Figure 8D). We then measured the cutin and wax contents of 22 DAP fruits in the ‘22-1’ and ‘30-1’ varieties using GC-MS. As shown in Figure 8E and Table S5, cutin content in ‘30-1’ was slightly higher than that of ‘22-1’, whereas wax content in ‘30-1’ was significantly lower than that in ‘22-1’. Ethyl stearate and 9- and 12-diepoxy, a dominant composition, accounted for 20.14% and 19.08% of the cutin in ‘30-1’ and ‘22-1’, respectively. Tetradecanedioic acid, monomethyl ester, (Z)-18-octadec-9-enolide, and 13-docosenamide, (Z)- also accounted for a large proportion of cutin (Table S5). Among the compositions of wax, benzene (1-pentyloctyl), phthalic acid, butyl dodecyl ester, 9-octadecenamide, (Z)-, hexanedioic acid, bis(2-ethylhexyl) ester, 2,2′-methylenebis, 6-tert-butyl-4 methylphenol, O,O′-bis(trimethylsilyl), bis(2-ethylhexyl) phthalate, 1-monopalmitin, 2TMS derivative, and n-hentriacontane displayed significant differences across ‘22-1’ and ‘30-1’. The content of all these compounds, except for n-hentriacontane, was higher in ‘22-1’ than in ‘30-1’ (Figure 8F). n-Tritriacotane played a dominant role in the wax context of ‘30-1’ but not in ‘22-1’. The 1-Monopalmitin and 2TMS derivatives accounted for the largest proportion of wax content in ’22-1’ (Table S5).

### 3.11. Identification of SmKCS Gene Family in Eggplant

As wax may contribute to fruit brightness and KCS is the rate-limiting enzyme of FA elongation, we further identified the entire SmKCS gene family in eggplants. A total of 21 candidate genes were identified using the blastp method in NCBI and the hidden Markov model (HMM) search program (PF08392.12). The length of SmKCS proteins ranged from 204 to 530 aa, with an average length of 426 aa and an average molecular weight of 47897.2 kda. The pI values ranged from 6.31 to 10 (Table 4).

### 3.12. Phylogenetic Analysis of SmKCS Proteins

**Figure 9:**
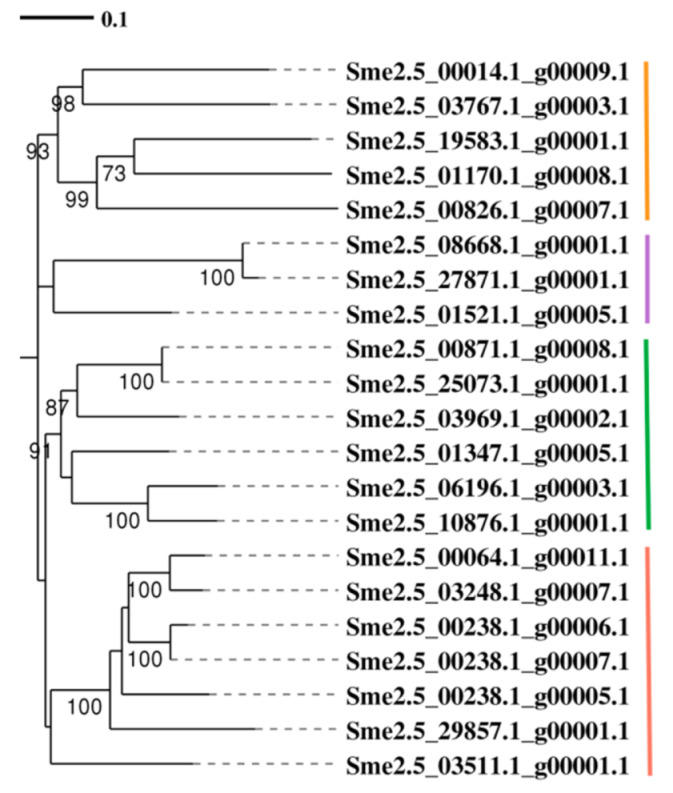
Phylogenetic tree of the SmKCS protein family in eggplant. The phylogenetic tree was constructed using the neighbor-joining method in MEGA7.0. The different groups are indicated with different colors.

To study the relationships between eggplant SmKCS proteins, we constructed a phylogenetic tree with the SmKCS protein sequences using MEGA7.0. As shown in Figure 9, SmKCS proteins were categorized into four subgroups. Among the 21 SmKCS proteins, 5 belong to subgroup I, 3 belong to subgroup II, 6 belong to subgroup III, and 7 belong to subgroup IV. The four SmKCS genes we mentioned above displayed significant differences in RNA-Seq. Among them, Sme2.5_00826.1_g00007.1 and Sme2.5_00014.1_g00009.1 are classified in group I. Sme2.5_01347.1_g00005.1 and Sme2.5_29857.1_g00001.1 are classified in group III and group IV, respectively.

### 3.13. Structure and Conserved Motif Analyses of the SmKCS Gene Family

**Figure 10:**
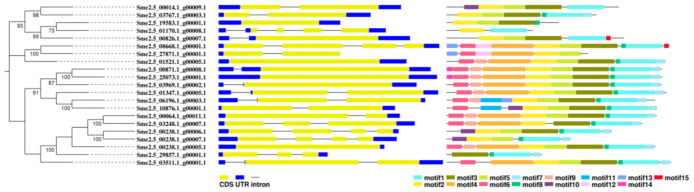
The intron–exon structure and conserved motifs of eggplant SmKCS genes according to their phylogenetic relationships. Lengths of introns, exons, and motifs of each SmKCS gene are shown as proportions.

To examine the gene structure diversity of SmKCS genes in eggplants, we analyzed their exon–intron organization. The four SmKCS genes contained no introns, five SmKCS genes contained two introns, three SmKCS genes contained three introns, and one gene contained four introns. The remaining eight genes each contained one intron. Sme2.5_00826.1_g00007.1 and Sme2.5_00014.1_g00009.1 contained one intron, while Sme2.5_01347.1_g00005.1 and Sme2.5_29857.1_g00001.1 contained two introns (Table S6 and Figure 10).

To further understand the structure of KCS genes in eggplants, conserved motifs were analyzed using the MEME tool. As shown in Figure 10 and Table S7, the 21 KCS genes contained 15 motifs. Motif 2 existed in other 20 KCS genes except Sme2.5_29857.1_g00001.1. Motif 1 is present in 19 genes except Sme2.5_19583.1_g00001.1 and Sme2.5_27871.1_g00001.1. Motifs 3 and 5 are present in 18 genes. Motif 8 existed in 17 genes. Sme2.5_00826.1_g00007.1 and Sme2.5_00014.1_g00009.1 both contained five motifs. Sme2.5_01347.1_g00005.1 contained nine motifs and Sme2.5_29857.1_g00001.1 contained four motifs (Figure 10).

### 3.14. qPCR Verification

**Figure 11:**
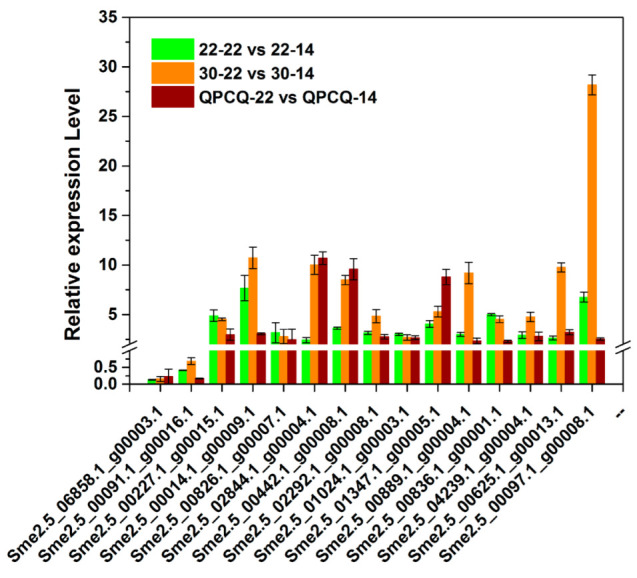
Verification of the expression of selected DEGs by qRT-PCR.

In order to validate the accuracy of RNA-seq data, we chose 15 genes related to wax biosynthesis, metabolism, and transportation, which were mentioned above in the 22 vs. 14 groups for qRT-PCR analysis (Table 3 and Figure 11). In total, 2 genes were downregulated and the other 13 genes were upregulated in the 22 vs. 14 groups. These results of the qRT-PCR analysis were similar to those of the RNA-seq data.

## 4. Discussion

Fruit brightness is an important commodity trait that affects consumer consumption. Research on fruit brightness has mainly focused on plants such as cucumbers, tomatoes, *Arabidopsis*, and navel oranges, but seldom on eggplants. We selected three varieties of eggplant with different brightness levels to investigate the dynamics and differential expression of genes using RNA-Seq. Across the 18 samples, the Q20 and Q30 of the reads were higher than 97% and 92%, respectively (Table S2), reflecting high sequencing reliability. The average mapping ratio of the samples to the reference genome was 82% (Table S3) and was comparable among the different samples.

To further illustrate the potential function of the DEGs, we conducted GO and KEGG pathway enrichment analyses. After analyzing the most significantly enriched 30 pathways in KEGG pathway analysis, we found that ‘biosynthesis of unsaturated fatty acids’, ‘fatty acid biosynthesis’, ‘fatty acid elongation’, ‘fatty acid metabolism’, ‘fatty acid degradation’, ‘cutin, suberine, and wax biosynthesis’, and ‘ABC transporter’, which are related to biosynthesis and metabolism, and ‘transport of cutin and wax’, represented a significant portion of the pathways (Figure 4D–F and Figure 5E–H). This result was consistent with that of previous studies. One study on tomato mutants showed that glossiness and increased stiffness in fruit peels are associated with cutin deficiency [31]. Petit et al. [11] found that a more complex cuticle architecture could be responsible for fruit brightness. In *Arabidopsis*, wax load or an alteration in wax compounds contributes to stem glossiness [12]. The SEM images obtained in this study (Figure S5 and Figure 8A–C) indicate that ‘22-1’ has a thicker and more irregular cuticle, which may have contributed to the dull appearance of ‘22-1’ fruits.

**Figure 12:**
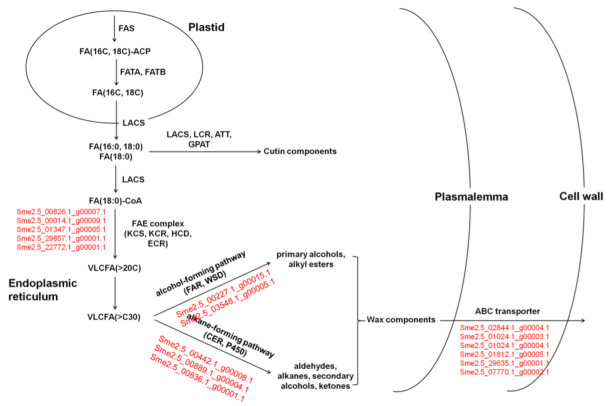
Hypothetical biosynthetic pathways for cutin monomers and wax constituents in fruit peel of eggplant. Genes with red color are candidate DEGs in hypothetical biosynthetic pathways.

We identified pathways related to cutin and wax in the three 22 vs. 14 groups. In total, 24 DEGs were selected as candidate genes using a Venn plot (Figure 6A). The selected genes encode proteins including LACS, KCS, ECR, WSD, and FAR. Two genes, which encode KCS and ABCC proteins, were downregulated at different fruit development stages among the different eggplant varieties. ECR, which constitutes one of the FAE complexes, regulates the fourth step of the reduction reaction in FA elongation. *TSC13/YDL015* was the first ECR-type gene isolated from yeast and is related to the synthesis of sphingolipids and the elongation of VLCFA [32]. The *CER10* gene, which encodes the ECR protein, displays a homologous relationship with *TSC13* in *A. thaliana*. Loss of function of the *CER10* gene can lead to a decrease in leaf cuticle wax, seed triacylglycerol, and sphingolipids [32]. *Sme2.5_22772.1_g00001.1*, which encodes the ECR protein, was downregulated in the 22 DAP vs. 14 DAP groups in our study (Figure 6C and Table 3). In contrast, *Sme2.5_02292.1_g00008.1*, which encodes a homologous protein of at5g57800, was upregulated in the 22 vs. 14 groups. *At5g57800* is also referred to as *CER3* and plays an important role in the alkane synthesis pathway [33]. Overexpression of the *CER1* gene increases the content of very long-chain alkanes and reduces the permeability of the cuticle, thereby reducing the non-stomatal loss of water and enhancing drought tolerance in *Arabidopsis* [34]. *CER1* interacts with the *WAX2*, *CER3*, and *CYTB5* genes. The interaction of these genes in yeast can convert very long-chain acyl-CoAs into very long-chain alkanes through redox reactions [35]. KCS catalyzes the polymerization of malonyl-CoA and long-chain acyl-CoA. Malonyl-CoAs and long-chain acyl-CoAs exhibit strict substrate specificity and their types determine the rate of the cyclic reaction and the acyl chain length of the final acyl-CoA products [36]. In *Arabidopsis*, two groups of *KCS* genes have been identified; the FAE1 *KCS* gene group, of which a total of 21 have been discovered so far, and the *ELO KCS* gene group. Currently, four of these genes have been annotated, but their functions have not yet been studied. Among them, *KCS1*, *KCS2*, *KCS6*, *KCS9*, and *KCS20* have been reported to regulate wax biosynthesis [36,37,38,39,40,41,42]. In our study, the expression of four *KCS* genes was significantly different between the three 22 vs. 14 groups (Figure 6C and Table 3). The expression of *Sme2.5_29857.1_g00001.1*, which encodes KCS6, also displayed significantly differential expression in groups 30-14 vs. 22-14, 30-22 vs. 22-22, QPCQ-14 vs. 22-14, and QPCQ-22 vs. 22-22. The ABC transporter located on the plasma membrane achieves the transportation of wax components across the plasma membrane. Five DEGs (three genes being upregulated and two genes being downregulated) belonging to the ABC transporter family were identified in three, 22 vs. 14 groups (Figure 6C and Table 3). *Sme2.5_07770.1_g00002.1*, which encodes ABC transporter C family member 8, was downregulated at different fruit development stages among the three eggplant varieties. Based on the above analyses, we suggest that ‘30-1’ and ‘QPCQ’ may affect wax synthesis by affecting the elongation of fatty acids and the export of wax components. The reduced wax content subsequently affects the cuticle structure of the peel, thereby generating a glossy appearance of the fruit (Figure 12).

Studies in recent years have found that TFs play an important role in regulating growth and development in plants. Mutation of the *Glossy 1* gene in maize causes cuticle wax on the leaves to nearly disappear. Its homologous genes *cer1* and *wax2* mutants in *Arabidopsis* also displayed cuticle wax defects on the stem [36,43]. In *Arabidopsis*, *MAH1* was identified to catalyze the conversion of alkanes into secondary alcohols and ketones [44]. WIN1/SHN1, a TF related to wax biosynthesis, directly or indirectly activates the transcription of genes encoding enzymes in cutin and wax biosynthesis. Overexpression of *WIN1* increases wax content and improves drought tolerance in *Arabidopsis* [45]. Arabidopsis TFs MYB16 and MYB106 participate in cuticle development [46]. MYB96 regulates the expression of *KCS1*, *KCS2*, *KCS6*, *WSD1*, *CER2*, and *KCR1* genes [47]. MYB30, MYB41, and CFL1 act as positive regulators in cutin and wax biosynthesis and cuticle development [48,49,50]. In our present research, 41 TFs displayed significant differences in fruit development. Among them, seven genes belonged to the MYB family (Figure 7C). After protein interaction analysis using the string website, we found that MYB30 (Sme2.5_00097.1_g00008.1) may participate in cuticle formation by interacting with KCS6 (Sme2.5_29857.1_g00001.1) and CER4 (Sme2.5_03548.1_g00005.1) (Figure 7B). Sme2.5_00097.1_g00008.1 may influence wax formation by interaction with KCS6 and CER4, and this needs further verification in the future.

## 5. Conclusions

This study selected three eggplant varieties, ‘22-1’, ‘30-1’, and ‘QPCQ’, with different fruit brightness levels for comparative transcriptomic analysis. After DEG enrichment analyses of GO and KEGG, we found that many pathways and genes related to cuticle cutin and wax content significantly differed between varieties. In total, 24 DEGs encoding proteins, such as KCS, ECR, FAR, and ABC transporters, were selected as candidate genes regulating the wax synthesis and transport in the 22-22 vs. 22-14, 30-22 vs. 30-14, and QPCQ-22 vs. QPCQ-14 groups. Two DEGs encoding KCS and ABC transporter proteins were commonly downregulated in the 30-14 vs. 22-14, 30-22 vs. 22-22, QPCQ-14 vs. 22-14, and QPCQ-22 vs. 22-22 groups. SEM images and measurements of cutin and wax content across the three eggplant varieties indicated that ‘22-1’ exhibited a thicker cuticle and higher wax content than those of the other two varieties. We further identified and analyzed the KCS gene family, which encodes the rate-limiting enzyme of FA elongation in wax synthesis in eggplant. These results will provide insight into the molecular mechanisms of fruit brightness in eggplants and allow further perspectives to be considered when crop breeding.

## Figures and Tables

**Table 1:** SNP variant type summary.

Sample	A-G	C-T	Transition	A-C	A-T	C-G	G-T	Transversion	Total
22-1-14-1	18,464	18,369	36,833	5698	6145	4105	5747	21,695	58,528
22-1-14-2	19,034	18,866	37,900	5841	6319	4208	5816	22,184	60,084
22-1-14-3	17,944	17,728	35,672	5464	5948	3973	5524	20,909	56,581
22-1-22-1	19,262	19,241	38,503	5913	6516	4364	5962	22,755	61,258
22-1-22-2	17,514	17,474	34,988	5409	5914	3998	5452	20,773	55,761
22-1-22-3	20,370	20,289	40,659	6255	6810	4492	6344	23,901	64,560
30-1-14-1	16,194	16,451	32,645	4951	5597	3756	5077	19,381	52,026
30-1-14-2	16,661	16,602	33,263	5062	5737	3777	5222	19,798	53,061
30-1-14-3	17,317	17,187	34,504	5195	5929	3848	5378	20,350	54,854
30-1-22-1	17,060	17,071	34,131	5183	5872	3764	5351	20,170	54,301
30-1-22-2	17,316	17,299	34,615	5178	5871	3814	5313	20,176	54,791
30-1-22-3	18,755	18,678	37,433	5686	6322	4094	5825	21,927	59,360
QPCQ-14-1	13,173	13,135	26,308	4058	4481	2862	4125	15,526	41,834
QPCQ-14-2	14,314	14,021	28,335	4378	4772	3086	4448	16,684	45,019
QPCQ-14-3	14,479	14,465	28,944	4419	4931	3135	4528	17,013	45,957
QPCQ-22-1	14,146	13,985	28,131	4304	4826	3063	4352	16,545	44,676
QPCQ-22-2	15,405	15,446	30,851	4696	5252	3351	4759	18,058	48,909
QPCQ-22-3	15,608	15,552	31,160	4762	5283	3398	4876	18,319	49,479

A-G: number of SNP for A-G variation. C-T: number of SNP for C-T variation. Transition: number of SNP for A-G and C-T variations. A-C: number of SNP for A-C variation. A-T: number of SNP for A-T variation. C-G: number of SNP for C-G variation. G-T: number of SNP for G-T variation. Transversion: number of SNP for A-C, A-T, C-G, and G-T variations.

**Table 2:** The number of pathways related to cutin and wax in groups 22 DAP vs. 14 DAP.

Pathways	22-22 vs. 22-14		30-22 vs. 30-14		QPCQ-22 vs. QPCQ-14	
	UP	DOWN	UP	DOWN	UP	DOWN
Biosynthesis of unsaturated fatty acids	9	2	1	3	1	1
Cutin, suberine, and wax biosynthesis	11	5	7	4	12	4
Fatty acid biosynthesis	9	3	1	3	2	0
Fatty acid degradation	5	2	5	3	5	1
Fatty acid elongation	6	5	4	4	4	3
Fatty acid metabolism	14	5	2	5	3	2
ABC transporters	16	3	6	3	7	3
SUM	48	18	22	16	28	11

**Table 3:** The expression level and annotation of DEGs related to cutin and wax in groups 22 DAP vs. 14 DAP.

GENE ID	Up/Down-Regulation	Annotation
Sme2.5_00826.1_g00007.1	Up	3-ketoacyl-CoA synthase 3
Sme2.5_00014.1_g00009.1	Up	3-ketoacyl-CoA synthase 20
Sme2.5_01347.1_g00005.1	Up	3-ketoacyl-CoA synthase 11-like
Sme2.5_29857.1_g00001.1	Down	3-ketoacyl-CoA synthase 6-like
Sme2.5_22772.1_g00001.1	Down	trans-2-enoyl-CoA reductase
Sme2.5_00227.1_g00015.1	Up	O-acyltransferase WSD1-like
Sme2.5_00442.1_g00008.1	Up	Cytochrome P450 77A1
Sme2.5_00889.1_g00004.1	Up	cytochrome P450 86A8
Sme2.5_00836.1_g00001.1	Up	cytochrome P450 86A22
Sme2.5_02292.1_g00008.1	Up	protein ECERIFERUM 3-like
Sme2.5_02154.1_g00006.1	Up	pleiotropic drug resistance protein 1
Sme2.5_08011.1_g00001.1	Up	pleiotropic drug resistance protein 1-like
Sme2.5_06858.1_g00003.1	Down	3-ketoacyl-CoA thiolase 2
Sme2.5_04239.1_g00004.1	Up	acyl-coenzyme A oxidase 4
Sme2.5_00625.1_g00013.1	Up	acyl-activating enzyme 16
Sme2.5_03548.1_g00005.1	Down	fatty acyl-CoA reductase 3-like
Sme2.5_00091.1_g00016.1	Down	putative elongation of fatty acids protein
Sme2.5_01164.1_g00001.1	Up	omega-hydroxypalmitate O-feruloyl transferase
Sme2.5_02990.1_g00002.1	Down	omega-hydroxypalmitate O-feruloyl transferase
Sme2.5_01024.1_g00003.1	Up	ABC transporter B family member 2
Sme2.5_01024.1_g00004.1	Up	ABC transporter B family member 2
Sme2.5_02844.1_g00004.1	Up	ABC transporter G family member 32
Sme2.5_29635.1_g00001.1	Down	ABC transporter G family member 4
Sme2.5_01812.1_g00005.1	Down	ABC transporter C family member 15

**Table 4:** The SmKCS proteins in eggplant.

Gene ID	MW (kda)	pI	Protein Length (aa)
Sme2.5_19583.1_g00001.1	29,582.9	10	265
Sme2.5_00826.1_g00007.1	47,212.4	8.45	419
Sme2.5_27871.1_g00001.1	37,796.3	9.19	334
Sme2.5_00871.1_g00008.1	57,046.7	9.12	509
Sme2.5_03969.1_g00002.1	56,934.8	9.45	508
Sme2.5_25073.1_g00001.1	57,046.7	9.12	509
Sme2.5_00014.1_g00009.1	45,569.2	8.37	407
Sme2.5_01347.1_g00005.1	58,512.9	9.37	519
Sme2.5_01521.1_g00005.1	57,632.5	9.69	516
Sme2.5_00064.1_g00011.1	55,893.1	9.25	496
Sme2.5_08668.1_g00001.1	59,843.1	9.24	527
Sme2.5_03767.1_g00003.1	39,626.2	6.31	354
Sme2.5_03248.1_g00007.1	52,167.6	9.06	462
Sme2.5_00238.1_g00006.1	43,558.2	8.89	390
Sme2.5_00238.1_g00007.1	32,572.5	7.81	294
Sme2.5_00238.1_g00005.1	55,707.6	9.42	493
Sme2.5_03511.1_g00001.1	59,793.8	9.27	530
Sme2.5_06196.1_g00003.1	55,317.6	9.58	493
Sme2.5_10876.1_g00001.1	56,023.5	9.44	496
Sme2.5_01170.1_g00008.1	22,573.9	8.49	204
Sme2.5_29857.1_g00001.1	25,430.2	9.56	225

## Data Availability

The data have been submitted to NCBI SRA database with accession number PRJNA721241.

## Supplementary Materials

The following supporting information can be downloaded at: https://www.mdpi.com/article/10.3390/foods11162506/s1, Figure S1: Distributions of SNPs in different groups. ‘Up2k’ and ‘Down2k’ indicate regions within 2000 bp of upstream and downstream of a gene, respectively; Figure S2: Distributions of Indels in different groups. ‘Up2k’ and ‘Down2k’ indicate regions within 2000 bp of upstream and downstream of a gene, respectively; Figure S3: GO functional enrichment analyses of DEGs. GO functional enrichment analyses of DEGs in groups 30-22 vs 30-14 (A), QPCQ-22 vs QPCQ-14 (B), 30-14 vs 22-14 (C), 30-22 vs 22-22 (D), QPCQ-14 vs 22-14 (E), and QPCQ-22 vs 22-22 (F). GO functional enrichment analyses of up- and down-regulated genes in groups 30-22 vs 30-14 (G), QPCQ-22 vs QPCQ-14 (H), 30-14 vs 22-14 (I), 30-22 vs 22-22 (J), QPCQ-14 vs 22-14 (K), and QPCQ-22 vs 22-22 (L); Figure S4: Transcription factors family classification; Figure S5: Scanning electron microscopy images of 14 and 22 DAP fruits peel from ‘22-1’, ‘30-1’, and ‘QPCQ’; Table S1: The primers used in qRT-PCR; Table S2: Summary of transcriptome sequencing data; Table S3: Summary of reads mapping to the reference genome; Table S4: New transcript type statistics; Table S5: Cutin and wax content in varieties ‘22-1’ and ‘30-1’; Table S6: The intron–exon structure of eggplant SmKCS genes; Table S7: Motifs in SmKCS.
