# Supplementary material for: Comparative Transcriptome Analysis Reveals Gene Expression Differences in Eggplant (Solanum melongena L.) Fruits with Different Brightness
Source: Foods. 2022 Aug 19;11(16):2506. doi: 10.3390/foods11162506 (PMC9407171; doi:10.3390/foods11162506)

Figure S1. Distributions of SNPs in different groups. ‘Up2k’ and ‘Down2k’ indicate regions within 2000 bp of upstream and downstream of a gene, respectively.

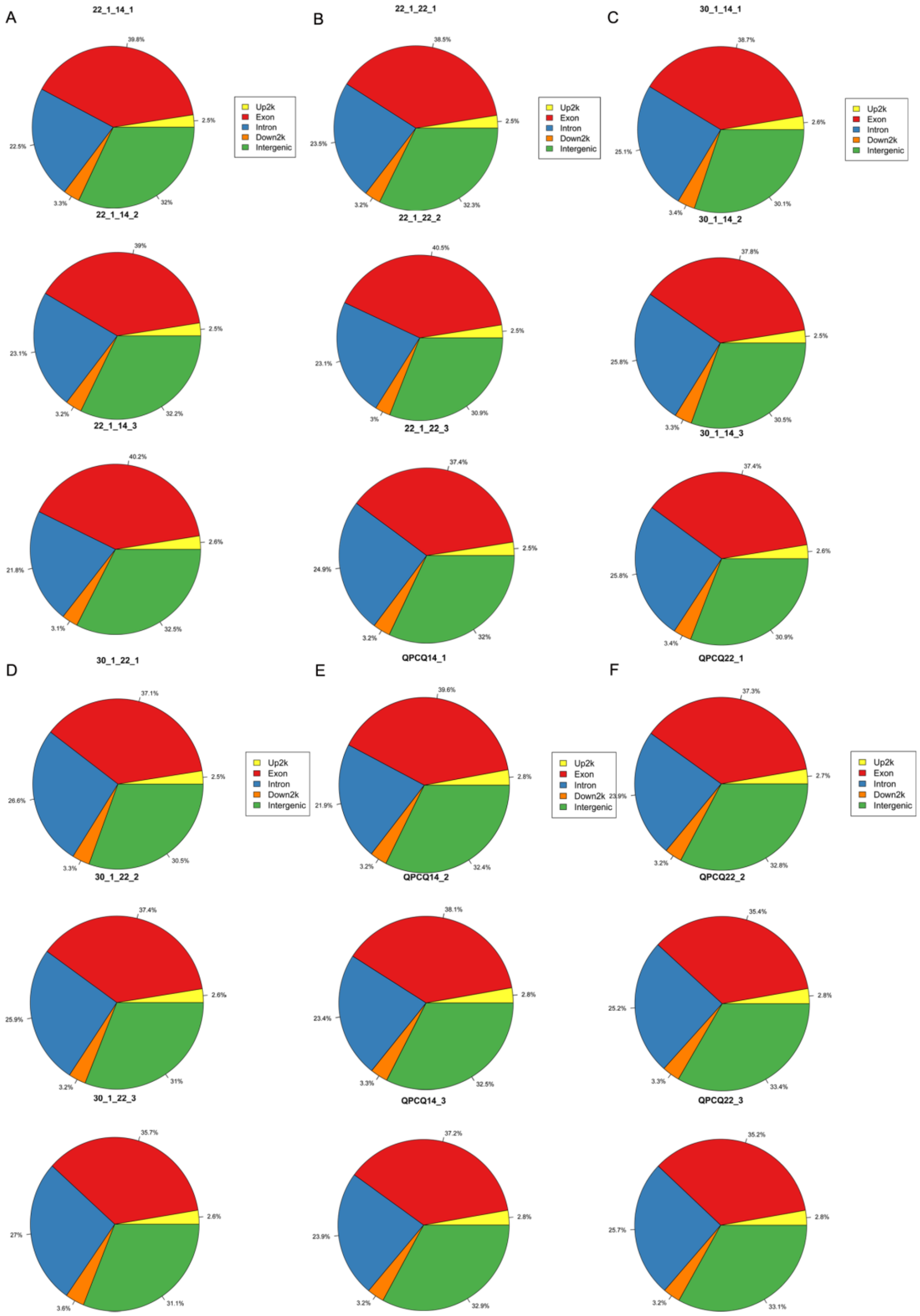

Supplement: Supplementary file 1 [file foods-11-02506-s001.zip › supplymentary files/Figure S1.pdf]
