# Supplementary material for: Comparative Transcriptome Analysis Reveals Gene Expression Differences in Eggplant (Solanum melongena L.) Fruits with Different Brightness
Source: Foods. 2022 Aug 19;11(16):2506. doi: 10.3390/foods11162506 (PMC9407171; doi:10.3390/foods11162506)

Figure S3. GO functional enrichment analyses of DEGs. GO functional enrichment analyses of DEGs in groups 30-22 vs 30-14 (A), QPCQ-22 vs QPCQ-14 (B), 30-14 vs 22-14 (C), 30-22 vs 22-22 (D), QPCQ-14 vs 22-14 (E), and QPCQ-22 vs 22-22 (F). GO functional enrichment analyses of up- and down-regulated genes in groups 30-22 vs 30-14 (G), QPCQ-22 vs QPCQ-14 (H), 30-14 vs 22-14 (I), 30-22 vs 22-22 (J), QPCQ-14 vs 22-14 (K), and QPCQ-22 vs 22-22 (L).

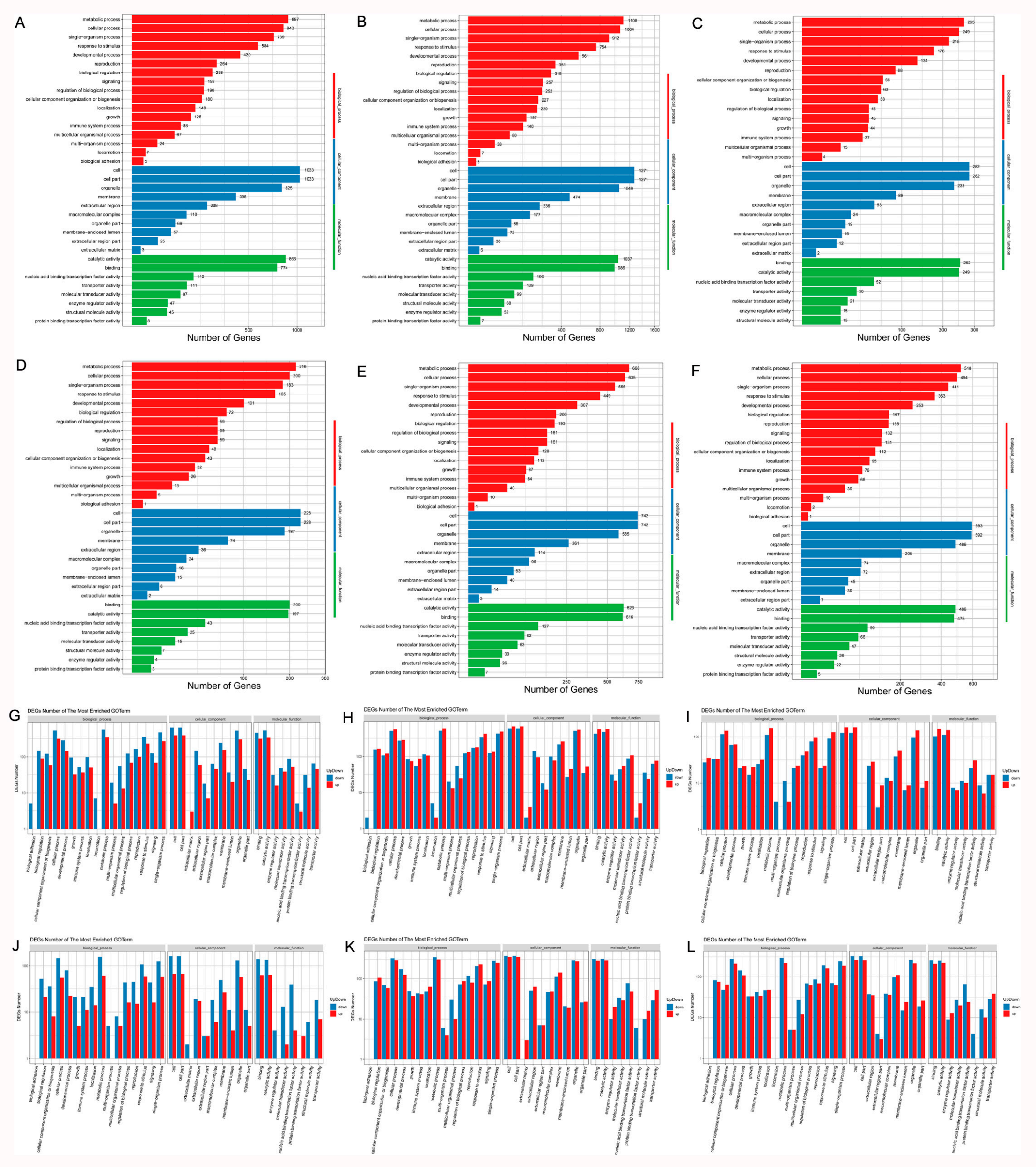

Supplement: Supplementary file 1 [file foods-11-02506-s001.zip › supplymentary files/Figure S3.pdf]
