# Supplementary material for: Comparative Transcriptome Analysis Reveals Gene Expression Differences in Eggplant (Solanum melongena L.) Fruits with Different Brightness
Source: Foods. 2022 Aug 19;11(16):2506. doi: 10.3390/foods11162506 (PMC9407171; doi:10.3390/foods11162506)
Supplement: Supplementary file 1 [file foods-11-02506-s001.zip › supplymentary files/Table S2.pdf]

Table S2. Summary of transcriptome sequencing data.

Sample	Reads No.	Bases (bp)	N (%)	Q20 (%)	Q30 (%)
22-1-14-1	53,559,100	8,033,865,000	0.000976	97.63	93.53
22-1-14-2	57,117,194	8,567,579,100	0.000965	97.35	92.86
22-1-14-3	46,862,498	7,029,374,700	0.000984	97.6	93.38
$22\text{-}14_{means}$	52,512,931	7,876,939,600	0.000975	97.53	93.26
22-1-22-1	55,130,286	8,269,542,900	0.000948	97.60	93.37
22-1-22-2	43,022,976	6,453,446,400	0.000974	97.36	92.84
22-1-22-3	52,100,136	7,815,020,400	0.000968	97.78	93.81
$22\text{-}22_{means}$	50,084,466	7,512,669,900	0.000963	97.58	93.34
30-1-14-1	47,513,304	7,126,995,600	0.000959	97.39	92.95
30-1-14-2	48,214,656	7,232,198,400	0.000945	97.70	93.68
30-1-14-3	50,543,298	7,581,494,700	0.000929	97.64	93.58
$30\text{-}14_{means}$	48,757,086	7,313,562,900	0.000944	97.58	93.40
30-1-22-1	45,277,480	6,791,622,000	0.000999	97.71	93.65
30-1-22-2	49,611,898	7,449,284,700	0.000948	97.70	93.64
30-1-22-3	55,853,278	8,377,991,700	0.000981	97.42	93.12
$30\text{-}22_{means}$	50,247,552	7,539,632,800	0.000976	97.61	93.47
QPCQ-14-1	49,358,380	7,403,757,000	0.000983	97.45	93.01
QPCQ-14-2	49,344,156	7,401,623,400	0.000992	97.63	93.5
QPCQ-14-3	50,258,728	7,538,809,200	0.000963	96.1	90.25
$QPCQ\text{-}14_{means}$	49,653,755	7,448,063,200	0.000979	97.06	92.25
QPCQ-22-1	46,173,508	6,926,026,200	0.000967	97.51	93.18
QPCQ-22-2	54,924,390	8,238,658,500	0.001003	97.44	93.16
QPCQ-22-3	54,333,748	8,150,062,200	0.000979	97.39	92.98
$QPCQ\text{-}22_{means}$	51,810,549	7,771,582,300	0.000983	97.45	93.11
