# Supplementary material for: Comparative Transcriptome Analysis Reveals Gene Expression Differences in Eggplant (Solanum melongena L.) Fruits with Different Brightness
Source: Foods. 2022 Aug 19;11(16):2506. doi: 10.3390/foods11162506 (PMC9407171; doi:10.3390/foods11162506)
Supplement: Supplementary file 1 [file foods-11-02506-s001.zip › supplymentary files/Table S3.pdf]

Table S3. Summary of reads mapping to the reference genome.

Sample	Total clean reads	Total mapping ratio	Uniquely mapping ratio
22-1-14-1	49,769,196	84.63%	81.55%
22-1-14-2	53,194,304	85.21%	82.14%
22-1-14-3	43,711,392	85.25%	82.25%
22-14$_{means}$	48,891,631	85.03%	81.98%
22-1-22-1	51,422,872	85.24%	81.27%
22-1-22-2	40,125,720	85.59%	81.12%
22-1-22-3	48,588,758	84.90%	80.96%
22-22$_{means}$	46,712,450	85.24%	81.12%
30-1-14-1	44,350,782	84.68%	81.60%
30-1-14-2	45,110,846	85.30%	81.90%
30-1-14-3	47,128,700	85.47%	82.31%
30-14$_{means}$	45,530,109	85.15%	81.94%
30-1-22-1	42,192,216	84.67%	80.53%
30-1-22-2	46,276,246	84.87%	80.24%
30-1-22-3	52,061,164	83.89%	80.18%
30-22$_{means}$	46,843,209	84.48%	80.32%
QPCQ-14-1	46,039,256	85.76%	82.53%
QPCQ-14-2	46,024,156	85.13%	82.13%
QPCQ-14-3	46,790,404	84.16%	81.05%
QPCQ-14$_{means}$	46,284,605	85.02%	81.90%
QPCQ-22-1	43,034,552	85.91%	80.90%
QPCQ-22-2	51,130,350	86.31%	81.86%
QPCQ-22-3	50,532,460	85.87%	81.93%
QPCQ-22$_{means}$	48,232,454	86.03%	81.56%
