# Supplementary material for: Comparative Transcriptome Analysis Reveals Gene Expression Differences in Eggplant (Solanum melongena L.) Fruits with Different Brightness
Source: Foods. 2022 Aug 19;11(16):2506. doi: 10.3390/foods11162506 (PMC9407171; doi:10.3390/foods11162506)
Supplement: Supplementary file 1 [file foods-11-02506-s001.zip › supplymentary files/Table S4.pdf]

Table S4. New transcript type statistics

Total novel transcript	Coding transcript	Noncoding transcript	Novel gene
30,726	26,158	4,568	26,158
