# Supplementary material for: Comparative Transcriptome Analysis Reveals Gene Expression Differences in Eggplant (Solanum melongena L.) Fruits with Different Brightness
Source: Foods. 2022 Aug 19;11(16):2506. doi: 10.3390/foods11162506 (PMC9407171; doi:10.3390/foods11162506)
Supplement: Supplementary file 1 [file foods-11-02506-s001.zip › supplymentary files/Table S6.pdf]

Table S6. The intron-exon structure of eggplant *SmKCS* genes.

Gene ID	CDS Length (bp)	UTR	Intron	Exon
Sme2.5_03969.1_g00002.1	1527	316	1	2
Sme2.5_00238.1_g00007.1	885	443	3	4
Sme2.5_00238.1_g00005.1	1482	207	0	1
Sme2.5_00238.1_g00006.1	1173	165	3	4
Sme2.5_19583.1_g00001.1	798	457	0	1
Sme2.5_00871.1_g00008.1	1530	579	1	2
Sme2.5_01170.1_g00008.1	615	307	4	5
Sme2.5_01521.1_g00005.1	1551	389	0	1
Sme2.5_01347.1_g00005.1	1560	305	2	3
Sme2.5_27871.1_g00001.1	1002	53	1	2
Sme2.5_00064.1_g00011.1	1491	206	1	2
Sme2.5_06196.1_g00003.1	1482	227	2	3
Sme2.5_03248.1_g00007.1	1389	254	2	3
Sme2.5_29857.1_g00001.1	678	137	2	3
Sme2.5_03511.1_g00001.1	1593	321	2	3
Sme2.5_00826.1_g00007.1	1260	515	1	2
Sme2.5_10876.1_g00001.1	1491	158	1	2
Sme2.5_08668.1_g00001.1	1584	216	3	4
Sme2.5_03767.1_g00003.1	1065	311	1	2
Sme2.5_25073.1_g00001.1	1530	725	0	1
Sme2.5_00014.1_g00009.1	1224	458	1	2
