# Supplementary figures and images for: Comparative Transcriptome Analysis Reveals Gene Expression Differences in Eggplant (Solanum melongena L.) Fruits with Different Brightness
Source: Foods. 2022 Aug 19;11(16):2506. doi: 10.3390/foods11162506 (PMC9407171; doi:10.3390/foods11162506)

Figure S4. Transcription factors family classification.

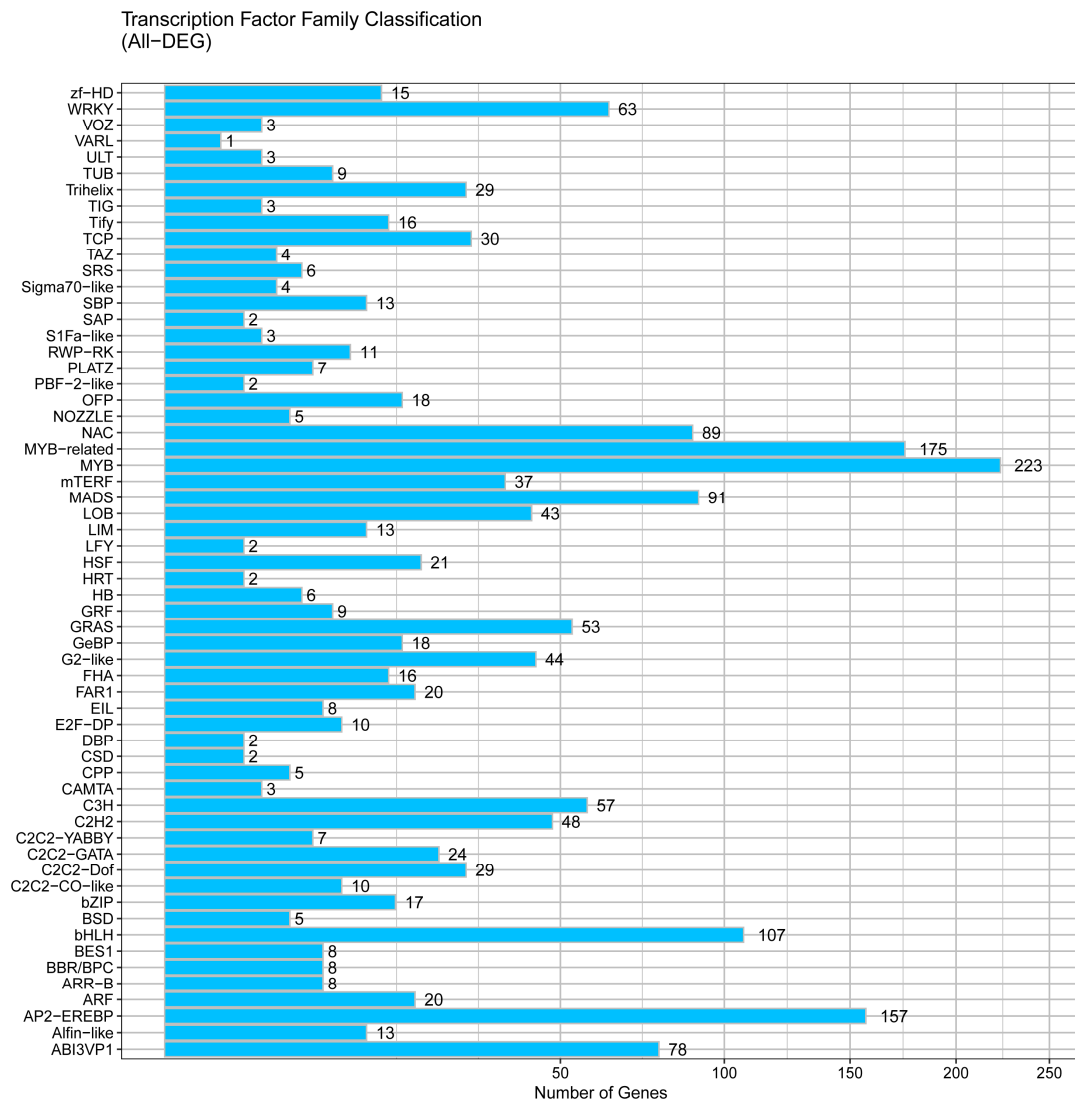

Supplement: Supplementary file 1 [file foods-11-02506-s001.zip › supplymentary files/Figure S4.pdf]

Figure S5. Scanning electron microscopy images of 14 and 22 DAP fruits peel from ‘22-1’, ‘30-1’, and ‘QPCQ’.

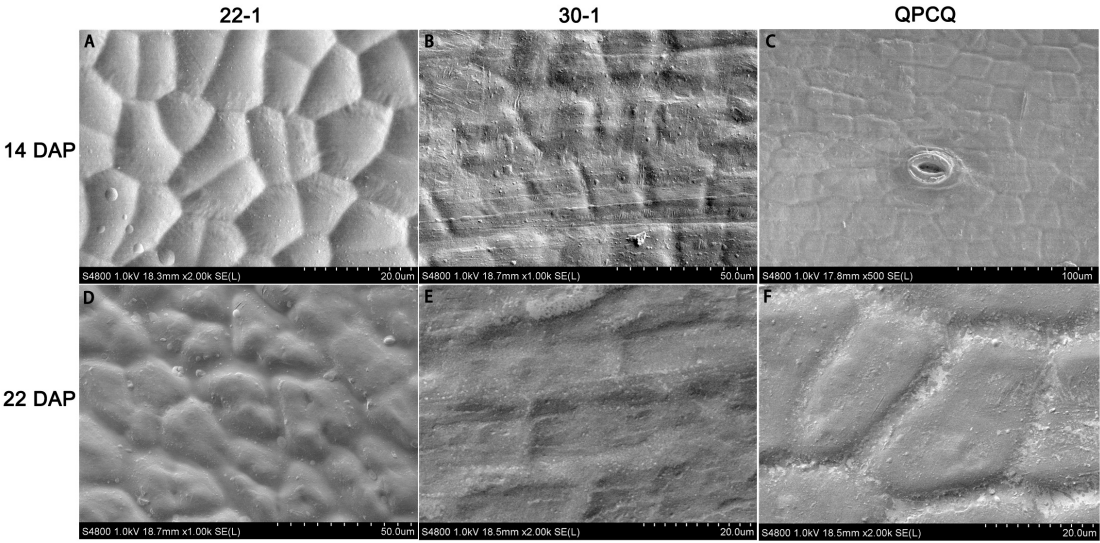

Supplement: Supplementary file 1 [file foods-11-02506-s001.zip › supplymentary files/Figure S5.pdf]
